# P/Q Type Calcium Channel Cav2.1 Defines a Unique Subset of Glomeruli in the Mouse Olfactory Bulb

**DOI:** 10.3389/fncel.2018.00295

**Published:** 2018-09-04

**Authors:** Martina Pyrski, Mahbuba Tusty, Eugenia Eckstein, Livio Oboti, Diego J. Rodriguez-Gil, Charles A. Greer, Frank Zufall

**Affiliations:** ^1^Center for Integrative Physiology and Molecular Medicine, Saarland University, Homburg, Germany; ^2^Department of Neuroscience and Department of Neurosurgery, School of Medicine, Yale University, New Haven, CT, United States

**Keywords:** olfactory subsystem, olfactory bulb, olfactory epithelium, voltage-gated calcium channel, Cav2.1α-1a subunit, *Cacna1a*, synaptic localization, olfactory glomerulus

## Abstract

Voltage-gated calcium (Cav) channels are a prerequisite for signal transmission at the first olfactory sensory neuron (OSN) synapse within the glomeruli of the main olfactory bulb (MOB). We showed previously that the N-type Cav channel subunit Cav2.2 is present in the vast majority of glomeruli and plays a central role in presynaptic transmitter release. Here, we identify a distinct subset of glomeruli in the MOB of adult mice that is characterized by expression of the P/Q-type channel subunit Cav2.1. Immunolocalization shows that Cav2.1+ glomeruli reside predominantly in the medial and dorsal MOB, and in the vicinity of the necklace glomerular region close to the accessory olfactory bulb. Few glomeruli are detected on the ventral and lateral MOB. Cav2.1 labeling in glomeruli colocalizes with the presynaptic marker vGlut2 in the axon terminals of OSNs. Electron microscopy shows that Cav2.1+ presynaptic boutons establish characteristic asymmetrical synapses with the dendrites of second-order neurons in the glomerular neuropil. Cav2.1+ glomeruli receive axonal input from OSNs that express molecules of canonical OSNs: olfactory marker protein, the ion channel Cnga2, and the phosphodiesterase Pde4a. In the main olfactory epithelium, Cav2.1 labels a distinct subpopulation of OSNs whose distribution mirrors the topography of the MOB glomeruli, that shows the same molecular signature, and is already present at birth. Together, these experiments identify a unique Cav2.1+ multiglomerular domain in the MOB that may form a previously unrecognized olfactory subsystem distinct from other groups of necklace glomeruli that rely on cGMP signaling mechanisms.

## Introduction

The mammalian olfactory system exhibits a highly complex organization that comprises several anatomically segregated chemoreceptive organs including the main olfactory epithelium (MOE), the vomeronasal organ (VNO), and the Grueneberg ganglion (GGN). Each of these organs contains functionally distinct groups of sensory neurons, or olfactory subsystems, that are differentiated based on their signal transduction mechanisms, the chemosensory cues they detect, and the axonal connections they establish to target specific regions in the olfactory bulb of the forebrain (Zufall and Munger, 2001; Munger et al., 2009; Bear et al., 2016). Sensory neurons located within the MOE and GGN project their axons to the main olfactory bulb (MOB) whereas vomeronasal sensory neurons (VSNs) project to a specific nucleus located posterior to the dorsal MOB known as the accessory olfactory bulb (AOB). A complete understanding of the organization of the olfactory system is essential for understanding the processing of chemosensory information and the role that each subsystem plays in odor-guided behaviors (Zufall and Munger, 2016).

The MOB of the mouse comprises ∼3,600 glomeruli (Richard et al., 2010) which form a sensory map on the surface of the bulb (Mori and Sakano, 2011). An olfactory glomerulus is a complex neuropil that receives axonal input from a distinct population of olfactory sensory neurons (OSNs) and contains the first synaptic connections of the olfactory pathway formed by presynaptic OSN boutons and post-synaptic dendrites of mitral and tufted cells or local interneurons (Shepherd et al., 2004; Wachowiak and Shipley, 2006). An estimate of the number of OSN synapses per glomerulus is ∼260,000, assuming 26 synapses per axon (Klenoff and Greer, 1998) and an average number of OSNs expressing a given odorant receptor (OR) gene of ∼10,000 (Bressel et al., 2016). The vast majority of MOB glomeruli receive sensory input from canonical OSNs. These employ a cAMP signaling cascade, express a given OR, and the axons from OSNs expressing the same OR genes coalesce into two or a few glomeruli (Ressler et al., 1994; Vassar et al., 1994; Firestein, 2001; Treloar et al., 2002; Richard et al., 2010; Bressel et al., 2016). The mouse MOB also comprises > 50 Taar+ glomeruli receiving input from OSNs that express a single receptor of the trace-amine associated receptor (Taar) family (14 members) and seem to rely on classical cAMP signal transduction (Liberles and Buck, 2006; Li and Liberles, 2016). Most Taar+ OSNs project to glomeruli located in the dorsal center of the olfactory bulb (Pacifico et al., 2012). Furthermore, a small number of OSNs rely on cGMP signaling mechanisms (Zufall and Munger, 2010). These include the GC-D+ OSNs which express the receptor guanylate cyclase GC-D (Fülle et al., 1995; Juilfs et al., 1997; Meyer et al., 2000) and are involved in the social transmission of food preference (Leinders-Zufall et al., 2007; Munger et al., 2010). GC-D+ OSNs have their axonal targets in 9–12 glomeruli of the “necklace” region encircling the caudal MOB (Juilfs et al., 1997; Leinders-Zufall et al., 2007). Interestingly, sensory neurons of the Grueneberg ganglion (GGNs), which express the receptor guanylate cyclase GC-G (Fleischer et al., 2009; Liu et al., 2009), also have their axonal targets in the MOB necklace region, although within nine distinct glomeruli (Matsuo et al., 2012; Bumbalo et al., 2017). A recently discovered subpopulation of OSNs (known as type B cells) that contain the ion channel Trpc2 and the soluble guanylate cyclase Gucy1b2 also use cGMP signaling to detect low environmental oxygen (Omura and Mombaerts, 2014, 2015; Bleymehl et al., 2016). The glomerular targets of these sensory neurons are located on the posteroventral surface of the MOB, in proximity to the necklace glomeruli (Omura and Mombaerts, 2014, 2015). Together, these results have revealed a surprisingly complex spatial and molecular organization of axonal projection targets in the mouse olfactory bulb representing the neural and genetic architecture of vertebrate olfaction (Bear et al., 2016).

Here, we present evidence for the existence of a novel multiglomerular domain in the mouse MOB that may represent a previously unrecognized olfactory subsystem. This subsystem is differentiated by the expression of the voltage-activated calcium (Cav) channel subunit Cav2.1 (also known as α1A, encoded by the gene *Cacna1a* in mice). Cav channels produce locally restricted intracellular Ca^2+^ transients in response to action potential firing at the active zone of presynaptic terminals (Catterall, 2011; Kamp et al., 2012). Of the three structurally and functionally related families (Cav1–Cav3) identified in the mammalian central nervous system, the Cav2 family members P/Q- (Cav2.1), N- (Cav2.2), and R-type (Cav2.3) conduct Ca^2+^ currents that initiate synaptic transmission. Neurotransmitter release at central synapses is primarily mediated by the high-voltage activated channels Cav2.1 and Cav2.2 (Catterall, 2011). In the olfactory system, both subunits have been identified in the olfactory mucosa using RNA techniques and Western blot analyses (Shiraiwa et al., 2007; Ibarra-Soria et al., 2014). Presynaptic Cav2.2 protein is present in the vast majority of glomeruli of MOB and AOB (Weiss et al., 2014) and plays a central role in transmitter release of OSNs and VSNs (Isaacson and Strowbridge, 1998; Wachowiak et al., 2005; Weiss et al., 2014). In the present study, we show that Cav2.1 represents a second candidate for olfactory signal transmission within a distinct subset of MOB glomeruli. These results provide important new information for isolating a separate population of glomeruli and their corresponding chemosensory neurons and for understanding the functional significance of this organization for regulating excitability of peripheral afferents in the olfactory system.

## Materials and Methods

### Mice

All procedures were approved by the Institutional Animal Care and Use Committee of Saarland University and were in full accordance with the laws for animal experiments of the German government. Experiments were performed on mouse tissues derived from mice at different ages and of both sexes. For the developmental study, we used mice at embryonic day 18 (E18), postnatal day 1 (P1), P7, P14, and P21. Results from adult mice were obtained at 6–18 weeks of age. We used wild type mice (C57BL/6J, denoted as B6), OMP-GFP^+/-^ mice (B6; 129P2-Omp^tm3Mom^/MomJ, The Jackson Laboratory; stock# 006667) that were heterozygous for both OMP (olfactory marker protein) and GFP (green fluorescent protein) (Potter et al., 2001) Gucy2d-Mapt-lacZ mice (Leinders-Zufall et al., 2007), GCG-Cre-GFP mice denoted as GCG-GFP (line 52, kindly provided by Ivan Rodriguez, University of Geneva, Switzerland) (Matsuo et al., 2012), Gucy1b2-IRES-tauGFP, Trpc2-IRES-taumCherry, and Trpc2-IRES-taulacZ mice (Omura and Mombaerts, 2014, 2015). Mice were housed in micro-isolator cages on a reverse 12:12-h light/dark cycle with water and food available *ad libitum*.

### Olfactory Tissue Preparation

Mouse tissue preparation followed previously described methods (Weiss et al., 2011, 2014; Bolz et al., 2017). Mice were anesthetized [165 mg/kg body weight ketamine (Pharmacia GmbH, Berlin, Germany) and 11 mg/kg body weight xylazine (Bayer Health Care, Leverkusen, Germany)] and transcardially perfused with phosphate-buffered saline (PBS) pH 7.4, followed by 2 or 4% (w/v) paraformaldehyde in PBS (for Ncam2 antibody, 2% PFA was used, see below). Mice younger than 2 days were decapitated and fixed by immersion in 2 or 4% PFA prepared in PBS for 24 h instead. For cryostat sections, olfactory tissue was incubated in 30% sucrose in PBS at 4°C for 2 days, embedded in O.C.T. (Tissue-Tek), and snap-frozen in a dry ice/2-methylbutane bath. Frozen tissue sections (12–50 μm) were collected on a cryostat (HM525; Microm, Walldorf, Germany), thaw-mounted onto glass slides (Superfrost Plus, Polysciences), and stored at -80°C.

### RNAscope Fluorescence *in situ* Hybridization

Coronal MOE cryosections (14 μm) of adult B6 mice were subjected to RNAscope *in situ* hybridization (Wang et al., 2012) using the RNAscope Fluorescent Multiplex Detection Kit (ACD Biotechne) and specific *Cacna1a* probes. The procedure was performed according to the manufacturer’s recommendations. Sections were pretreated in 1 x citrate buffer pH 6.0 (DAKO, Germany) for 5 min at 98°C, rinsed in distilled water, dehydrated in 100% ethanol, air dried and treated with Protease III for 30 min at 40°C. Then, sections were rinsed in water and hybridized with the RNA scope target probe generated for mouse *Cacna1a* (ACD Biotechne) that was designed as channel 2 probe containing 20 ZZ structures targeting base pairs 5705-6727 of NM_007578.3^1^ distributing to exons 37–46. Two negative controls were run in parallel with the sample, the bacterial DapB probe supplied by the company (ACD Biotechne) and a negative control without any probe. Following hybridization (2 h, 40°C), sections were rinsed twice for 2 min in wash buffer (ACD Biotechne), and sequentially treated with amplification solutions at 40°C (30 min Amp1-FL, 15 min Amp2-FL, 30 min Amp3-FL) and fluorescence reagent (15 min, 40°C Amp4-altB), with intermitting washing steps between reagents at room temperature. Cell nuclei were stained with DAPI solution (ACD Biotechne) for 1 min, and tissue sections were mounted with DAKO fluorescence medium and stored for 12 h at 4°C. Fluorescence images were acquired on a Zeiss LSM 880 confocal microscope containing a 32-channel GaAsP-PMT and 2-channel PMT QUASAR detector.

### Reverse Transcription Polymerase Chain Reaction (RT-PCR)

Main olfactory epithelium tissue of two adult B6 mice was dissected, pooled, and then transferred to RNA*later*^®^ solution (Ambion). Total RNA extraction and cDNA synthesis were exactly as described previously (Pyrski et al., 2017). For polymerase chain reactions (PCR), we used 0.5 μl of cDNA, *Cacna1a*-specific primers, and the Phusion High Fidelity DNA polymerase (NEB) according to the manufacturer’s protocol. *Cacna1a* sequence information was retrieved from the Ensembl database (ENSMUSG00000034656). The *Cacna1a* mRNA contains 47 exons. Primers were chosen to amplify the region between exons 24–43. Forward (F) and reverse (R) primers in 5′–3′ orientation were F6-ex24: GGGGCCTATTTCCGTGAC, R3-ex38: GTCCATCCGCAGGAGTCTC, F8-ex37: CCTCATAGGGTTGCTTGCAAG, R7-ex43: CCATTTCTCGCATCTCCACAG, F9-ex34: CCAAATCACGGAGCACAATAAC (Eurofins Genomics, Ebersberg, Germany). Cycling parameters were 15 s at 98°C, followed by 39 cycles of (10 s at 98°C – 10 s at 60°C – 30 s at 72°C), and a final elongation (5 min at 72°C) prior to cooling samples to 4°C. Amplicon size in base pairs (bp) for each reaction was F6-ex24/R3-ex38: 1,731 bp; F8-ex37/R7-ex43: 607 bp; PCR-products were verified by gel electrophoresis on 1% agarose gels containing ethidium bromide, and by direct sequencing (Seqlab) of the purified amplicons using the primers listed above.

### Immunohistochemistry

All procedures were conducted at room temperature (20°C) except for incubations of tissue sections with primary antibodies that was done at 4°C. Tissue sections were incubated for 1 h in blocking reagent containing 4% normal horse serum (NHS, Vector Laboratories) and 0.3% Triton X-100 prepared in PBS, followed by incubation in primary antibody diluted in blocking reagent for at least 24 h. Primary antibodies and control peptides were Cav2.1 rabbit polyclonal (1:1000, #152103 Synaptic Systems) or a biotinylated version of that same antibody (1:1000, synthetized on request, Synaptic Systems); control peptide RDPDARRAWPGSPERAPGREGPYGRESEPQQRE (Genscript) corresponding to amino acids 856–888 of the mouse P/Q-type α-1a channel subunit Cav2.1, UniProt Id: P97445; β-galactosidase chicken polyclonal (1:500, cat. #ab9361, Abcam); Cnga2, rabbit polyclonal (1:500, Alomone cat. # APC-045); RFP rabbit polyclonal (1:2000, cat. #ABIN 129578, AK-Online); GFP chicken polyclonal (1:1000, cat. #ab13970, Abcam), Map2 chicken polyclonal (1:300, cat. #ab5392, Abcam); Ncam2 goat polyclonal (1:100, cat. #AF778, R&D Systems), OMP (1:3000, goat polyclonal; gift of F. Margolis, University of Maryland, College Park, MD, United States); Pde2a goat polyclonal (1:200, cat. #sc-17227, St. Cruz); Pde4a rabbit polyclonal (1:200, cat. #PD4-112ab, FabGennix); panTaar goat polyclonal (1:100, cat. #sc-54398, St. Cruz); vGlut1 mouse monoclonal (1:100, cat. #135511, Synaptic Systems); vGlut2 rabbit polyclonal (1:2000, cat. #135403, Synaptic Systems). Sections were washed 3 × 10 min in PBS and incubated with fluorescence-labeled secondary antibody or streptavidin conjugates (all Invitrogen) as follows. Alexa Fluor 488 donkey-anti-goat (1:1000, A-11055), Alexa Fluor 488 donkey-anti-chicken (1:1000, A-11039), Alexa Fluor 488 goat-anti-rabbit (1:1000, A-11034), Alexa Fluor 488 goat-anti-mouse (1:1000, A-11029), Alexa Fluor 555 donkey-anti-rabbit (1:1000, A-31572), Alexa Fluor 546 donkey-anti-goat (1:1000, A-11056); Alexa Fluor 647 donkey-anti-mouse (1:1000, A-31571), biotinylated goat-anti-rabbit (1:100, BA-1000, Vector Laboratories), Alexa 488-conjugated streptavidin (1:400; S-32354), Alexa Fluor 647 goat-anti-chicken (1:1000, A-21449), Alexa 546-conjugated streptavidin (1:400; S-11225). For the colocalization of Cav2.1 with a second primary antibody made in rabbit, we performed two sequential reactions with the biotinylated Cav2.1 antibody and fluorescence-conjugated streptavidin in the second reaction. Alternatively, we introduced a blocking step between reactions in which unbound rabbit IgG epitopes from the first reaction were blocked for 1 h with donkey-anti-rabbit Fab-fragments (1:50, Biomol, Rockland, ME, United States). Nuclei were stained with 2 μM Hoechst 33342 nuclear dye (Invitrogen) or with 5 μM Draq5 (BD Pharmingen) prior to cover slipping sections in fluorescence mounting medium (DAKO). The specificity of the immunostainings was verified by control experiments omitting the primary antibody or by incubation with pre-absorbed Cav2.1 antiserum (5 μg/ml blocking peptide).

### Whole-Mount Immunohistochemistry

Following transcardial perfusion with 4% PFA, olfactory bulbs were dissected and washed twice in PBS for 15 min prior to dehydration in graded methanol (25, 50, 100% methanol in PBS, 10 min each step). Olfactory bulbs underwent three freeze–thaw cycles for 30 min at -80°C and 10 min at room temperature. Following rehydration in graded methanol (50%, 25% methanol in PBS) for each 10 min, olfactory bulbs were washed in PBS for 15 min incubated in blocking buffer containing 0.5% Triton X-100 and 5% normal horse serum (Vector Laboratories) for 3 h at room temperature. Tissue was then incubated in primary antibody solution (see above) for 3 days at 4°C, washed three times in PBS for 1 h each, incubated in secondary antibody (1:1000) overnight at 4°C, and washed three times in PBS for 1 h each.

### Fluorescence Microscopy and Image Assembly

Fluorescence images of stained cryosections were acquired on a BX61 epifluorescence microscope attached to a DP71 camera (Olympus), or on a LSM 880/ConfoCor-3 confocal microscope (Zeiss). Confocal images are 1 μm digital images or Z-stacks presented as maximum intensity projections of 10–20 confocal sections, each 0.4 μm thick. Fluorescence images of olfactory bulb whole-mount preparations were acquired on a SZX16 epifluorescence binocular (Olympus). Images were assembled and adjusted in contrast and brightness using Photoshop Elements 10 (Adobe Photoshop).

### Olfactory Bulb 3D Reconstruction

The 3D reconstruction technique was as described previously (Luzzati et al., 2011). For the spatial representation of Cav2.1+ glomeruli in the MOB, serial coronal cryosections (50 μm) of adult OMP-GFP mice were subjected to Cav2.1 immunolabeling visualized with Alexa555-conjugated secondary antibody and nuclear staining using DAPI (4′, 6-diamidino-2-phenylindole, Vector Laboratories). Every section was acquired and digitized images were uploaded into Reconstruct software (Fiala, 2005). The 3D morphology of Cav2.1+ glomeruli was captured by 2D contour delineation that was verified by the presence of endogenous OMP-GFP fluorescence in each glomerulus and by the glomerular shape given by the nuclear staining of periglomerular cells. Import of the 3D assembly into the open source software Blender (Blender.org) allowed the editing of shading, transparency, and lighting.

### Immuno-Electron Microscopy

Processing for immuno-electron microscopy followed procedures we employ routinely (Weiss et al., 2011; Bartel et al., 2015). Adult (postnatal days 57–58) CD1 mice were euthanized with Euthasol and perfused using a low-/high-pH PFA fixation strategy (Treloar et al., 2002). This strategy was adopted after preliminary studies indicated that ultrastructural immunolocalization of the Cav2.1 antibody could be compromised in tissue perfused with fixatives containing glutaraldehyde. Tissue was initially perfused with 4% PFA in PBS (0.1 M phosphate buffer and 0.9% NaCl) at pH 6.5 for 5 min followed by 4% PFA in PBS at pH 10.5. Brains were removed and post-fixed overnight at 4°C in the second perfusate. Coronal vibratome sections of the MOB were free-floating immunostained using the Cav2.1 antibody as described above. Sections were incubated in biotin-conjugated goat anti-rabbit IgG secondary antibody (1:100, cat. #BA-1000, Vector Laboratories) for 1 h at room temperature. Sections were washed and incubated with ABC reagent (prepared by diluting both solution A and solution B at 1:50 in blocking buffer, Vector Laboratories) for 1 h at room temperature. Sections were washed again prior to a final rinse in PBS. Peroxidase activity was visualized by incubating tissue in 0.05% 3.3′-diaminobenzdine tetra hydrochloride (DAB), 0.005% H_2_O_2_ in TBS. The reaction was stopped in PBS and the tissue then incubated for 1 h in 2% glutaraldehyde in PBS. Sections were washed again (as above) and immediately processed for electron microscopy. Sections containing Cav2.1 immunolabeled glomeruli were re-embedded in EPON and sectioned at 70–90 nm on a Reichert Ultramicrotome prior to imaging in a JEOL 1200 electron transmission microscope.

## Results

### Cav2.1 Immunostaining Identifies a Unique Multiglomerular Domain in the Main Olfactory Bulb

**FIGURE 1:**
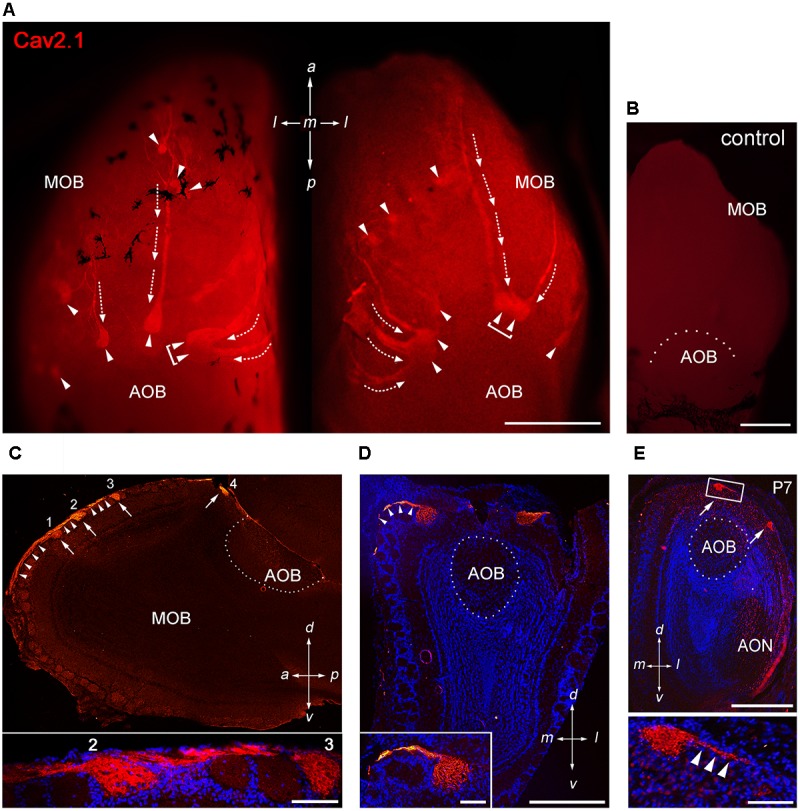
Cav2.1 staining reveals a unique subset of glomeruli in the MOB. **(A)** Cav2.1 immunoreactivity (red) exemplified in a whole-mount preparation of the left and right MOB (dorso-caudal view) of an adult B6 mouse. Cav2.1+ axon bundles (arrows) terminate into several individual glomeruli (arrowheads). Large Cav2.1+ glomeruli reside in the caudal aspect of the MOB, close to the accessory olfactory bulb (AOB). Frequently, two glomeruli reside next to each other (brackets). Staining is absent in the AOB. **(B)** Negative control reaction using peptide blocking is devoid of Cav2.1 staining (dorso-caudal view and right bulb). **(C)** Sagittal section (14 μm) at about the MOB midline depicting four individual Cav2.1+ glomeruli (arrows 1–4) and their afferent axonal fibers (arrowheads). The inset (bottom) shows a higher magnification of two glomeruli (2, 3) and axonal projections from the nerve layer of the MOB. Nuclei staining with Hoechst dye (blue) verifies glomerular boundaries. **(D)** Coronal section (14 μm) of the posterior MOB showing two large Cav2.1+ glomeruli medial and lateral to the AOB. The inset at the bottom depicts a higher magnification of the medial glomerulus. **(E)** Cav2.1+ glomeruli (arrows) are already evident at postnatal day 7 (P7) including afferent axons (arrowheads, inset, and bottom). **(A–E)** Orientations are as depicted by arrows indicating a, anterior; p, posterior; d, dorsal; v, ventral; l, lateral; m, medial; AON, anterior olfactory nucleus. Scale bars **(A–E)** overviews, 500 μm; **(C,D)** insets, 100 μm; **(E)** inset, 50 μm.

To assess whether the P/Q-type channel subunit Cav2.1 may represent a second calcium channel involved in synaptic transmission at the first olfactory synapse, we conducted a systematic immunohistochemical analysis of the olfactory bulb of adult B6 mice (**Figure 1**). We analyzed whole-mount preparations (*N* = 9 mice) and tissue sections taken at intervals along anterior-to-posterior and medial-to-lateral axes of the MOB (*N* = 13 mice) using a Cav2.1-specific antibody that has been established previously in Cav2.1 knockout mice (Nishimune et al., 2012; Maejima et al., 2013). Whole-mount staining of olfactory bulbs revealed robust Cav2.1 immunoreactivity in a small subset of glomeruli and their afferent axon bundles (**Figure 1A**) whereas negative control reactions using Cav2.1 antibody that was blocked with its cognate peptide lacked any immunoreactivity (**Figure 1B**). The Cav2.1+ glomeruli were mainly located in the dorsal and medial aspects of the MOB; ventral and lateral regions only occasionally showed labeled glomeruli. The most prominent Cav2.1+ glomeruli were situated at the dorsal-caudal border of the MOB encompassing 5–7 labeled glomeruli per bulb that surrounded the dorsal AOB in a semi-circular arrangement (**Figure 1A**). This part of the MOB contains the necklace glomerular region that is innervated by GC-D OSNs and GGNs both of which employ non-canonical second messenger pathways (Bear et al., 2016). However, Cav2.1+ glomeruli did not exhibit the necklace-typical “beads on a string” connectivity. Cav2.1+ glomeruli in the caudal MOB were rather large (≥100 μm diameter) compared to additional smaller ones (40–80 μm diameter) in the more anterior region of the dorsal MOB (**Figure 1A**). Frequently, we also observed small clusters of 2–3 neighboring Cav2.1 labeled glomeruli in the vicinity to the AOB (**Figure 1A**, brackets). However, in contrast to our previous analysis of the expression of N-type calcium channel Cav2.2 (Weiss et al., 2014), we did not detect Cav2.1+ glomeruli within the AOB (**Figures 1A,C,D**).

Interestingly, afferent OSN axon bundles appeared to coalesce early in the anterior MOB. In some instances, we observed thick axon bundles traveling for more than 500 μm across the dorsal MOB surface until terminating within posterior glomeruli (**Figure 1A**). Extending this observation, sagittal MOB sections showed that these axon bundles segregated onto several consecutive glomeruli along the anterior-to-posterior axis (**Figure 1C**). We also observed axon bundles projecting along the lateral and medial sites of the MOB to reach dorsal glomeruli located in the caudal MOB (**Figures 1A,D**). Examining the MOB during early postnatal development showed that the subset of Cav2.1+ glomeruli is already detectable as early as postnatal day 7 (**Figure 1E**).

### Quantitative 3D Reconstruction of Cav2.1+ Glomeruli

**FIGURE 2:**
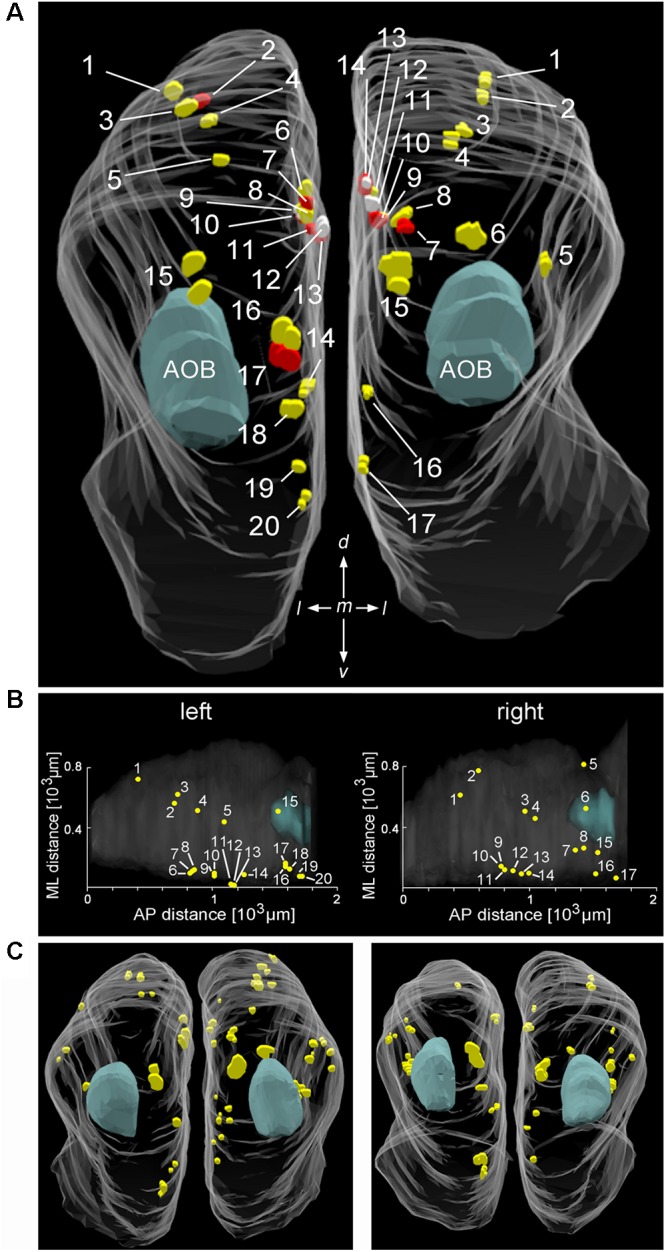
Distribution and position of Cav2.1+ glomeruli in the MOB. **(A)** Representative bilateral 3D reconstruction of the Cav2.1+ glomeruli in the MOB of an adult mouse (4 month, caudal view). Both left and right olfactory bulbs were collected as serial 50 μm sections from the same mouse. Closely adjacent glomeruli are differently colored (yellow, red, and white) to facilitate identification. The majority of Cav2.1+ glomeruli is located on the dorsal and medial MOB surface. The location of the AOB is as indicated in blue. **(B)** Dorsal view of the two MOBs shown in **(A)** illustrating the Cartesian localization of Cav2.1+ glomeruli in reference to their position along the medial-to-lateral (ML) axis and their relative position along the anterior-to-posterior (AP) extent of MOBs. **(C)** 3D reconstruction of the left and right MOB hemispheres of two additional mice (8 weeks) further illustrates the distribution of Cav2.1+ glomeruli in the dorsal and medial MOB.

To analyze Cav2.1 expression in the MOB in more detail, we generated glomerular maps using 3D reconstruction of serial MOB sections stained for Cav2.1. **Figures 2A,B** shows a reconstruction of the bulbs from a 4-month-old OMP-GFP mouse in which we identified 20 glomeruli in the left and 17 glomeruli in the right bulb. Reconstructions from two additional OMP-GFP mice are depicted in **Figure 2C**. Assembly of sections from these six bulbs revealed average numbers of 18 ± 1 Cav2.1+ glomeruli at the left and 21 ± 4 Cav2.1+ glomeruli at the right bulb hemisphere (mean ± SEM). Number and exact positions of labeled glomeruli varied somewhat between the left and right bulbs of individual mice and between individuals as such, but the overall topography of these glomerular domains was comparable between bulbs (**Figures 2A,C**). The majority of labeled glomeruli occupied the dorsal and medial aspects of the caudal half of the MOB (**Figures 2B,C**). Occasionally, we also detected labeled glomeruli in the ventral or lateral MOB.

Together, the results of **Figures 1**, **2** show that Cav2.1 immunolabeling reveals a subset of olfactory glomeruli in the MOB that defines a specific multiglomerular domain.

### Cav2.1 Localizes to Presynaptic OSN Axon Terminals

**FIGURE 3:**
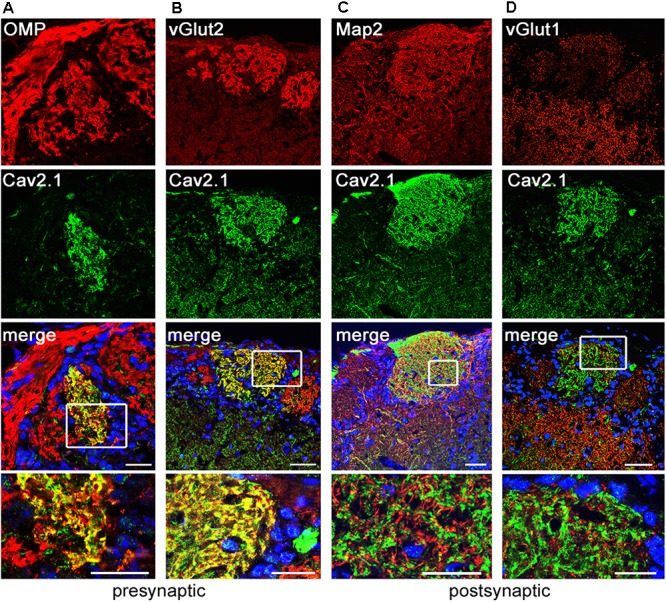
Cav2.1 is localized to presynaptic OSN axon terminals. Confocal images showing immunoreactivity (red) for **(A)** OMP, **(B)** vGlut2, **(C)** Map2, and **(D)** vGlut1 in combination with Cav2.1 (green) in coronal MOB sections of adult B6 mice. **(A,B)** The merged images illustrate that the presynaptic markers OMP and vGlut2 colocalize with Cav2.1 yielding the yellow fluorescence signal. **(C,D)** In contrast, the post-synaptic dendritic markers Map2 and vGlut1 do not colocalize with Cav2.1 in the glomerular neuropil. This is illustrated in the magnified merge at the bottom (boxed area) showing separate red and green fluorescence signals. Scale bars **(A–D)** 40 μm, inset magnifications, 20 μm.

We next examined whether glomerular Cav2.1 staining solely originates from the OSN presynaptic boutons or also from post-synaptic membranes of mitral and tufted cells that form synapses with OSN axon terminals in the glomerular neuropil. In addition to staining for OMP, we performed double-labeling immunohistochemistry for Cav2.1 in combination with several pre- and post-synaptic markers in MOB tissue sections of adult mice (**Figure 3**). Our results show that all Cav2.1+ glomeruli are also labeled for OMP indicating that Cav2.1 glomeruli receive axonal input from mature OSNs. Cav2.1 staining also colocalizes with the vesicular glutamate transporter vGlut2 (**Figures 3A,B**) that is selectively expressed in presynaptic OSN axon terminals (Gabellec et al., 2007; Richard et al., 2010). Thus, Cav2.1+ glomeruli receive axonal input from mature OMP+/vGlut2+ sensory neurons located in the MOE. By contrast, double-labeling for Cav2.1 and the microtubule-associated protein Map2 that identifies mitral cell dendrites showed no colocalization as illustrated by the separate red and green fluorescence signals, respectively (**Figure 3C**). Cav2.1 staining was also absent in dendrodendritic synapses established between mitral/tufted cells and local interneurons, as specified by colocalization with the vesicular glutamate transporter vGlut1 (**Figure 3D**).

**FIGURE 4:**
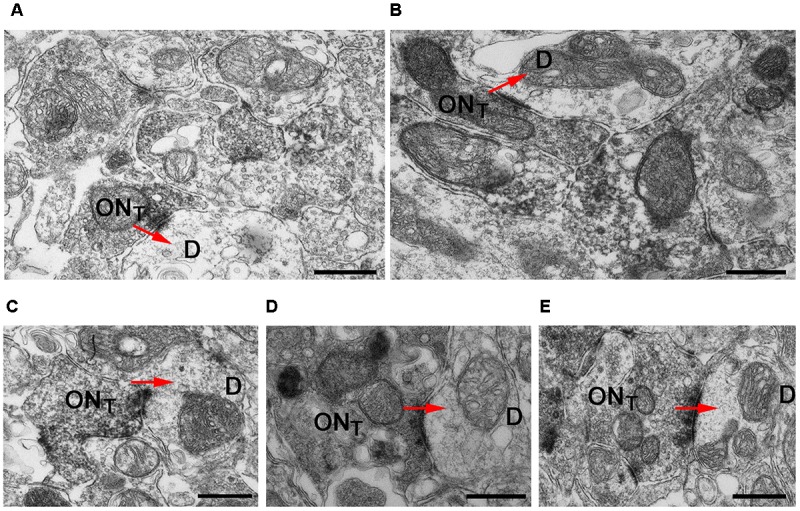
Cav2.1+ OSN axons form typical asymmetrical synapses with post-synaptic dendrites in glomeruli. **(A–E)** Examples of Cav2.1+ OSN terminals (ON_T_) establishing asymmetric synapses with unstained and electron lucent dendrites (D) are shown. Arrows (red) indicate the polarity of the synapses. Labeled axonal terminals were evident from the accumulation of the DAB precipitate among the presynaptic vesicles and membrane specializations. There was also a typical darkening of mitochondrial membranes in labeled terminals. In Cav2.1+ glomeruli, only ON_T_ are visible suggesting that Cav2.1 is homogeneously expressed by the axons innervating glomeruli. Scale bars: 500 nm.

To assess the presynaptic localization of Cav2.1 at a higher level of resolution and to evaluate the morphology of synapses formed by Cav2.1+ presynaptic boutons in more detail, we conducted immuno-electron microscopy. As illustrated by the different examples shown in **Figure 4**, OSN axon terminals showed robust immunoreactivity, and formed typical asymmetrical synapses with post-synaptic mitral cell dendrites that were devoid of any staining. Within a glomerulus, there was no evidence of unlabeled OSN terminals proximal to labeled OSN terminals, suggesting that Cav2.1 is homogeneously expressed by the axons innervating these glomeruli. Moreover, ultrastructural features of Cav2.1-labeled and unlabeled OSN terminals in neighboring glomeruli were undistinguishable. Vesicle density, organization of pre- and post-synaptic membrane specializations, and the distribution of mitochondria were altogether unremarkable (**Figure 4**). Thus, the localization of Cav2.1 to presynaptic axon terminals strongly implies a function of Cav2.1 in the signal transmission and transmitter release at the first olfactory synapse of these glomeruli.

### A Small Number of Cav2.1+ Glomeruli Express Ncam2

**FIGURE 5:**
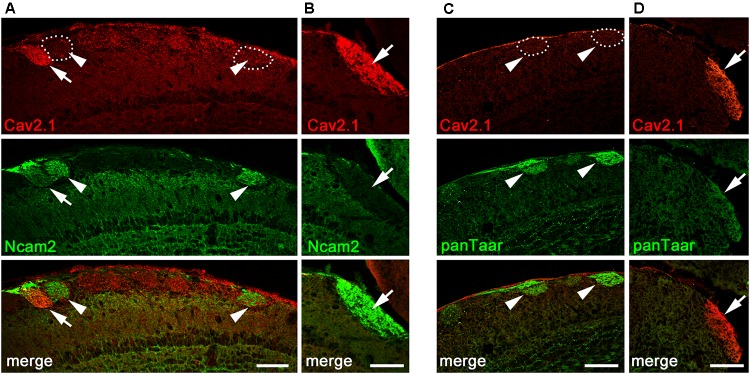
Cav2.1+ glomeruli and expression of neural adhesion molecules. **(A,B)** Confocal images of the dorsal-caudal MOB (sagittal plane) stained with Cav2.1 (red) and neural cell adhesion molecule Ncam2 (green). **(A)** The single Cav2.1+ glomerulus in the central-dorsal MOB exhibiting immunoreactivity for Ncam2 (arrow) represents a rare observation. Other Ncam2+ glomeruli in this area are devoid of Cav2.1 immunoreactivity (arrowheads). **(B)** Cav2.1+ glomeruli in the dorso-caudal MOB are Ncam2– (arrow) as depicted by the large glomerulus. **(C,D)** Double-labeling immunohistochemistry for Cav2.1 (red) and Taar (green) in the dorsal center **(C)** and dorso-caudal border **(D)** of the MOB. Cav2.1+ glomeruli are not labeled by the panTaar antibody used. Images are representatives of (*N* = 3) adult B6 mice, with *N* representing every second section per mouse. Scale bars: 100 μm.

The glomerular topography in the MOB is associated with the expression of neural cell adhesion molecules in sensory neurons located in specific zones of the olfactory epithelium. The neural cell adhesion molecule Ncam2 (also known as Ocam) defines the ventral and lateral MOE-to-MOB projections whereas dorsal and medial MOE-to-MOB projections are negative for Ncam2 (Yoshihara et al., 1997; Treloar et al., 2002; Walz et al., 2006). The dorsal and medial distribution of Cav2.1+ glomeruli led us to examine the expression of Ncam2 in adult B6 mice using double-labeling immunohistochemistry (**Figure 5**). We found that the majority of dorsal Cav2.1+ glomeruli were negative for Ncam2 whereas Ncam2+ glomeruli of the ventral and lateral MOB were devoid of Cav2.1 staining (**Figure 5A,B**). However, as an exception to the rule, we detected several Ncam2+ glomeruli in the dorsal MOB. Of these, about 2–3 glomeruli per bulb showed colocalization with Cav2.1 (*N* = 6 bulbs from three mice) (**Figure 5A**). It has been suggested previously that the dorsal glomeruli of the trace amine-associated receptor (Taar) subsystem, in particular those expressing Taar4-6, typically colocalize with Ncam2 (Johnson et al., 2012). To investigate whether glomeruli of the Cav2.1 and Taar subsystems may partially overlap, we conducted double-labeling experiments in MOB sections using Cav2.1 and a pan-specific antibody that detects a broad range of different Taar family members. However, we found that individual glomeruli in the dorsal MOB were singly stained for either panTaar (**Figure 5C**) or for Cav2.1 (**Figure 5D**) in the mice investigated (*N* = 3 bulbs from three different B6 mice), with individual Cav2.1+ glomeruli receiving axonal input from either Ncam2+ or Ncam2- OSNs.

### Cav2.1+ Glomeruli Express Transduction Molecules of Canonical OSNs

**FIGURE 6:**
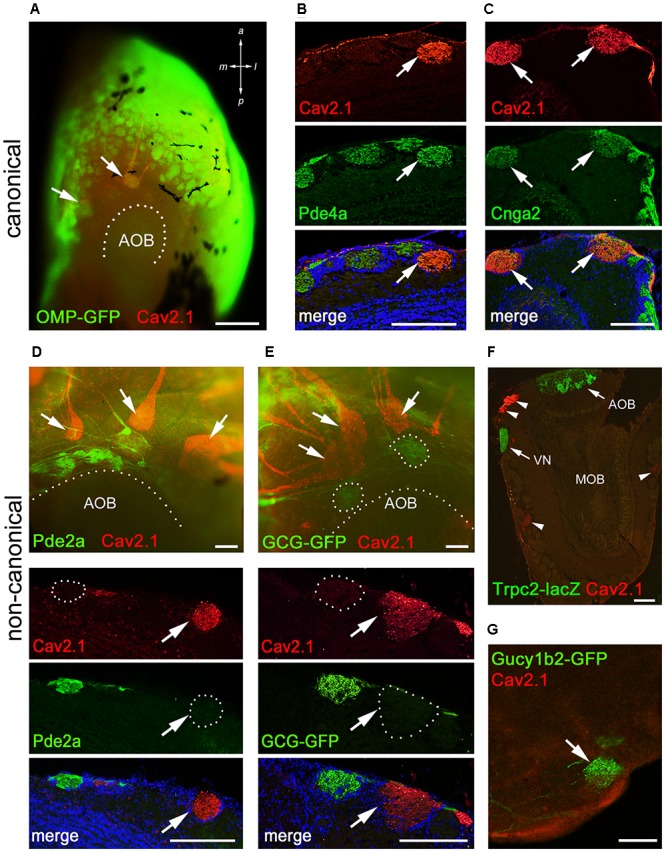
Cav2.1+ glomeruli receive afferent input from a specific subset of OSNs. **(A)** Cav2.1 immunoreactivity (red) and endogenous GFP fluorescence in a whole-mount MOB preparation (dorsal-caudal view) of an adult OMP-GFP mouse. Cav2.1 and GFP colocalize in individual glomeruli (arrows). Coronal MOB section (14 μm) showing that Cav2.1+ glomeruli (red) colocalize with Pde4a (green) **(B)** and Cnga2 (green) **(C)**. **(D,E)** Non-canonical markers are absent in Cav2.1+ glomeruli. **(D)** Whole-mount view (top) showing that Cav2.1+ glomeruli (arrows) reside anterior to the Pde2a+ glomeruli (green) and the AOB. Pde2a and Cav2.1 label separate sets of glomeruli (bottom, 14 μm coronal section). **(E)** Whole-mount view of the MOB (top) of a GCG-GFP mouse. Cav2.1+ glomeruli (red, arrows) reside anterior to the GFP+ glomeruli (green) that receive axonal input from GGNs. GCG-GFP+ and Cav2.1+ glomeruli are separate sets of glomeruli (bottom, 14 μm coronal section). **(F)** Coronal MOB section (14 μm) from a Trpc2-IRES-taulacZ mouse stained for Cav2.1 (red) and β-galactosidase (green). Both the AOB and the vomeronasal nerve (VN) but not the Cav2.1+ glomeruli (arrowheads) are positive for β-galactosidase. **(G)** Sagittal section (14 μm) of the caudal-ventral MOB from a Gucy1b2-IRES-tauGFP mouse. The single GFP+ (green) glomerulus shown is devoid of Cav2.1 staining (red). Hoechst nuclear dye (blue) defines glomerular boundaries in the merged images. Images are representatives of *N* ≥ 2 mice with *N* = every second section per mouse. Scale bars **(A)** 500 μm, **(B–E)** 100 μm, **(F)** 200 μm, **(G)** 50 μm.

Within the main olfactory system, a number of functionally different subsystems have been identified (Munger et al., 2009; Bear et al., 2016). To determine whether Cav2.1 glomeruli belong to any of the known subsystems, we conducted colocalization experiments with a variety of canonical and non-canonical markers in combination with Cav2.1. Immunostaining of MOB whole-mounts and tissue sections showed that Cav2.1+ glomeruli colocalize OMP and two typical elements of the canonical signal transduction pathway, the phosphodiesterase Pde4a and the cAMP-activated cation channel Cnga2 (**Figures 6A–C**). Thus, Cav2.1 glomeruli receive axonal input from mature sensory neurons of the MOE that seem to belong to the group of canonical OSNs in that they express at least two elements of the classical cAMP-mediated signal transduction cascade.

Because of the necklace-like arrangement of some Cav2.1+ glomeruli at the dorsal-caudal border of the MOB, we investigated the colocalization of Cav2.1 with other non-canonical signal transduction molecules. These included phosphodiesterase Pde2a that specifically labels necklace glomeruli targeted by GC-D OSNs of the MOE and the glomeruli targeted by the GGNs (Juilfs et al., 1997; Matsuo et al., 2012); the membrane guanylate cyclase GC-G present in GGNs (Fleischer et al., 2009; Liu et al., 2009); and the soluble guanylate cyclase Gycy1b2 and the Trpc2 cation channel that mediate signal transduction in type B cells of the MOE (Bleymehl et al., 2016). We found that Cav2.1+ glomeruli were devoid of Pde2a (**Figure 6D**). Whole-mount staining revealed that Cav2.1+ glomeruli were located in close proximity but always anteriorly to the Pde2a+ glomeruli (**Figure 6D**). There was also no colocalization between Cav2.1 and GFP using GCG-GFP mice that exclusively report on glomeruli innervated by GGNs in the MOB necklace region (Matsuo et al., 2012) (**Figure 6E**). Furthermore, colocalization experiments performed in Trpc2-IRES-taulacZ mice and Gucy1b2-IRES-taumCherry mice (Omura and Mombaerts, 2014, 2015) showed no colocalization with Cav2.1 glomeruli (**Figures 6F,G**). Together, these experiments demonstrate that the Cav2.1+ glomeruli do not belong to the olfactory subsystems formed by GC-D+ OSNs, GGNs, or Trpc2+ MOE (type A or type B) cells. Instead, Cav2.1+ glomeruli are characterized by the expression of OMP, Cnga2, and Pde4a.

### Cav2.1 Expression Defines a Distinct Subpopulation of OSNs

**FIGURE 7:**
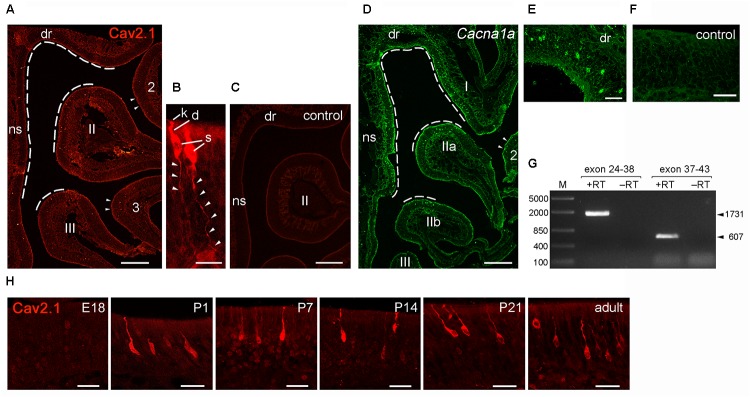
Identification of Cav2.1+ OSNs in the MOE. **(A)** Coronal MOE sections (14 μm) showing the left nasal cavity of an adult B6 mouse stained with Cav2.1. Cav2.1+ OSNs reside mainly in the MOE lining the dorsal roof (dr), nasal septum (ns), the dorsal-medial tip of endoturbinate II, and the tip of endoturbinate III (dashed lines). Few labeled OSNs are detectable at the tips of ectoturbinates 2 and 3. **(B)** Cav2.1 staining is present in OSN knob (k), dendrite (d), soma (s), and axon (arrowheads). To delineate axonal Cav2.1 staining, contrast and brightness was increased by 50%. **(C)** The peptide control reaction is devoid of Cav2.1 staining. **(D)** RNAscope fluorescence *in situ* hybridization for Cav2.1 mRNA (*Cacna1a*, green) in the MOE of an adult B6 mouse (coronal view, left nasal cavity) shows that the distribution of labeled OSNs is closely similar to that obtained by immunohistochemistry **(A)**. The dorsal roof, endoturbinate I, nasal septum, and the dorsal-medial tips of endoturbinates IIa and IIb (dashed lines) show labeled OSNs. Few labeled OSNs reside at the medial tip of ectoturbinate 2 (arrowheads). **(E)** Higher magnification shows hybridized OSNs at all depths of the epithelial layer. **(F)** The negative control reaction is devoid of labeling. **(G)** Agarose gel electrophoresis of the products obtained by RT-PCR using *Cacna1a*-specific primers and total RNA of adult mouse olfactory tissue. RT-PCR resulted in products (+RT) of the expected sizes using primers amplifying exons 24–38 (1,731 bp, arrowhead) and exons 37–43 (607 bp, arrowhead). Control reactions omitting reverse transcriptase (-RT) yielded no products. M, DNA size marker in base pairs as indicated at the left. **(H)** Magnifications of the dorsal MOE derived at different mouse ages stained with Cav2.1. Immunoreactivity for Cav2.1 (red) is absent at embryonic day 18 (E18). Cav2.1+ OSNs become visible at about postnatal day 1 (P1), and expression continues at P7, P14, P21 toward adulthood. Images are representatives of *N* ≥ 2 mice per age with *N* ≥ 10 sections per mouse. Scale bars **(A,C,D)** 200 μm, **(E)**; 50 μm; **(F,H)** 20 μm; **(B)** 10 μm.

Having identified Cav2.1 in a small subset of MOB glomeruli and their afferent axon bundles, we next focused on the corresponding neurons in the MOE. We performed immunohistochemistry for Cav2.1 on sections taken at intervals along the anterior-to-posterior extent of the MOE of adult B6 mice. As shown in **Figure 7**, robust Cav2.1 immunoreactivity was detected in a subpopulation of OSNs mainly situated in the medial and dorsal aspects of the posterior two thirds of the MOE. More specifically, Cav2.1+ OSNs occupied the epithelium lining the dorsal roof, the nasal septum, and the dorsomedial tip of endoturbinate II (**Figure 7A**). Fewer Cav2.1+ OSNs were detected in the dorsal tip of endoturbinate III and the medial aspect of ectoturbinate 2. Interestingly, this pattern is reminiscent to that of ORs. Based on the circumscribed mRNA expression of individual ORs, four dorso-ventral zones have been assigned along the anterior-to-posterior extent of the MOE, with zone 1 representing the most dorsal and medial aspects and zone 4 the most ventral and lateral aspects of the epithelium (Ressler et al., 1994). Referring to these zones, Cav2.1 expression was primarily detected in OSNs of zone 1 and the dorso-medial aspects of zones 2 and 3. Cav2.1 immunoreactivity depicted the typical bipolar OSN morphology and was present in the main subcellular compartments with strongly labeled knobs, dendrites, and somata (**Figure 7B**). OSN axons in the MOE were also stained but less so than the other subcellular compartments of the OSN (**Figure 7B**). Cav2.1+ OSNs were scattered throughout the depth of the MOE. The expression pattern was nearly bilaterally symmetrical between left and right nostrils and reproducibly observed among individual mice (*N* ≥ 3). Immunoreactivity was absent when omitting primary antibody and in peptide control reactions, where the Cav2.1 antiserum has been pre-absorbed with the cognate peptide (**Figure 7C**).

We obtained independent support for these results by RNAscope fluorescence *in situ* hybridization (**Figures 7D–F**). We used MOE tissue sections of adult B6 mice taken at anterior, medial and posterior positions of the epithelium and hybridized them with RNAscope probes specific for the 1 kb region spanning exons 37–46 of the *Cacna1a* mRNA. Our results showed strong hybridization signals in a subpopulation of OSNs that was scattered in the dorsal and medial MOE (**Figure 7D**). This expression pattern mirrored the pattern obtained by immunohistochemistry, and independently verified the specificity of the Cav2.1 antibody. The specificity of the hybridization signals was verified in control reactions that were devoid of any staining (**Figure 7F**). In addition, the hybridization region was verified by reverse transcription polymerase chain reaction (RT-PCR) using total RNA prepared from the nasal mucosa of adult B6 mice and gene-specific primers amplifying exons 24–43 of the *Cacna1a* mRNA (**Figure 7G**). Sequence analysis of the products obtained revealed 100% identity with the *Cacna1a* mRNA.

Cav channel expression at central nervous system neurons can be subject to developmental changes (Iwasaki et al., 2000; Mark et al., 2011) which may indicate a special requirement during periods of heightened plasticity. We examined Cav2.1 expression in the MOE at different time points during development. We analyzed coronal MOE tissue sections derived from B6 mice at embryonic day 18 (E18), postnatal day 1 (P1), P7, P14, and P21 (**Figure 7H**). At E18, the MOE was devoid of any Cav2.1 staining. However, at 1 day after birth we detected substantial Cav2.1 immunoreactivity in somata, dendrites, and dendritic knobs of OSNs. The number of labeled OSNs was relatively small at P1 but increased coinciding with age and epithelial thickness over the next 3 weeks of postnatal development. Cav2.1 expression in OSNs distributed in the dorsal and medial MOE was already pronounced at P7 (not shown), but overall we did not observe gross changes in the Cav2.1 expression pattern during the first 3 weeks of postnatal development (**Figure 7H**).

Together, our results show that Cav2.1 expression identifies a specific subpopulation of OSNs in the dorsal and medial MOB with an expression onset at about birth. This distribution is maintained by the Cav2.1+ glomeruli in a typical MOE-to-MOB topographic projection pattern.

### Cav2.1+ OSNs Express Markers of Canonical OSNs

**FIGURE 8:**
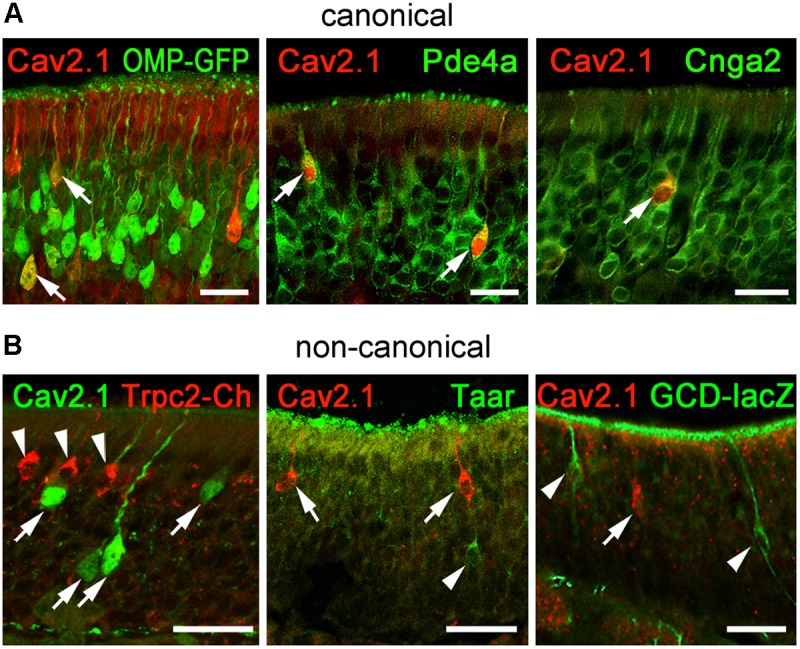
Cav2.1+ OSNs express the canonical markers OMP, Pde4a, and Cnga2. Coronal MOE sections (14 μm) of adult mice labeled for Cav2.1 and different canonical **(A)** and non-canonical **(B)** signal transduction molecules. **(A)** As shown at the left, Cav2.1 (red) and GFP (green) colocalize in mature OSNs (arrows) of OMP-GFP mice. Cav2.1+ OSNs (red) express Pde4a (green, middle, arrows) and Cnga2 (green, right, arrow). **(B)** Cav2.1 immunoreactivity (arrows) is absent in non-canonical OSNs such as those expressing Trpc2 (red, left, arrowheads), Taar (green, middle, arrowhead) or GC-D (green, right, arrowhead). Images are representatives of (*N* ≥ 2) mice with *N* = every 10th section along the anterior-to-posterior extent of the MOE in each mouse. Scale bars: 20 μm.

To gain further insight into the identity of Cav2.1+ OSNs and to verify our results obtained for Cav2.1+ glomeruli in the MOB, we performed immunohistochemical colocalization experiments on MOE tissue sections using several molecular markers. In addition to OMP whose expression coincides with OSN identity and maturation (Keller and Margolis, 1976), we examined expression of Pde4a and Cnga2. Furthermore, we analyzed components that define non-canonical OSNs such as GC-D OSNs, Taar+ OSNs, and Trpc2+ OSNs. These OSN subpopulations are distributed in regions of the MOE that partially overlap with those mapped for Cav2.1+ OSNs. Cav2.1 immunostaining in OMP-GFP reporter mice (Potter et al., 2001) showed that most Cav2.1+ OSNs are mature OSNs (**Figure 8A**) as indicated by the endogenous GFP fluorescence. Furthermore, Cav2.1 immunoreactivity clearly colocalized with both Pde4a and with Cnga2 (**Figure 8A**), demonstrating that Cav2.1+ OSNs express these components of canonical OSNs. By contrast, Cav2.1 showed no colocalization with any of the non-canonical components investigated (**Figure 8B**). For the colocalization with Trpc2, we analyzed MOE sections of Trpc2-IRES-taumCherry mice (Omura and Mombaerts, 2015) and antibodies directed against the fluorescence reporter. For GC-D, we used olfactory tissue sections from Gucy2d-Mapt-lacZ mice (Leinders-Zufall et al., 2007) and antibodies specific for β-galactosidase. Cav2.1 staining was also combined with the panTaar antibody. As shown in **Figure 8B**, Cav2.1+ OSNs were devoid of staining for Trpc2, Taar, and GC-D. Even if OSNs existed in close proximity to those neuron types, they always represented separately labeled OSN populations.

In summary, these results indicate that Cav2.1+ OSNs may represent a specialized subpopulation of canonical OSNs in the MOE that project to a unique subset of Cav2.1+ glomeruli in the MOB.

## Discussion

This study investigated the expression of the P/Q-type voltage-gated calcium channel subunit Cav2.1 in the mouse MOB and MOE using immunohistochemistry, immuno-electron microscopy, RT-PCR, and RNA scope *in situ* hybridization methodology. Our experiments provide several converging lines of evidence indicating that Cav2.1 represents a novel candidate for olfactory signal transmission in a previously unknown subset of MOB glomeruli. The main findings of this study are: (1) Cav2.1 expression in the MOB is limited to a unique subset of glomeruli mainly located in the dorso-caudal and medial aspects of each olfactory bulb. (2) Cav2.1 localizes to the presynaptic axon terminals of OSNs targeting these glomeruli. (3) Both Cav2.1 protein and its corresponding mRNA *Cacna1a* also localize to a defined subpopulation of OSNs in the dorsal and medial MOE, indicating a distinct MOE-to-MOB topography of Cav2.1+ OSNs. (4) Cav2.1 expression demarcates a previously unknown multiglomerular domain in the MOB, perhaps even a novel olfactory subsystem that is characterized by the expression of OMP, Cnga2, and Pde4a. (5) This system is distinct from several cGMP-dependent olfactory subsystems that express type-D and type-G receptor guanylate cyclases or Trpc2 and the soluble guanylate cyclase Gucy1b2.

**FIGURE 9:**
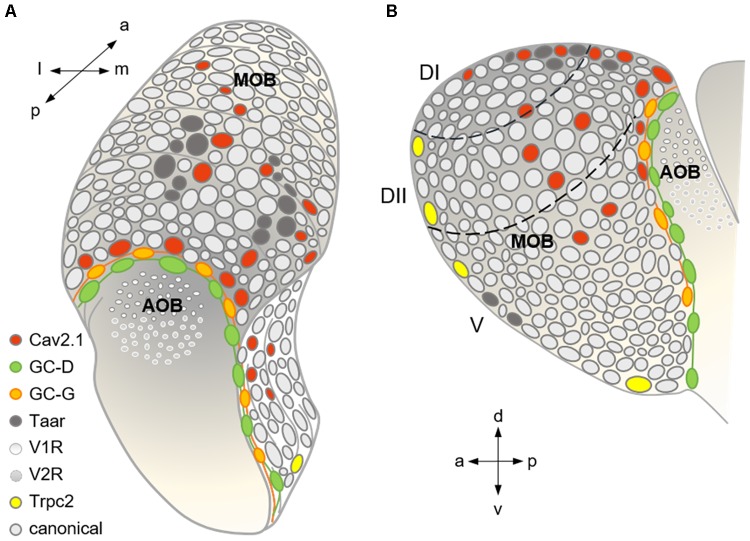
Summary scheme showing the organization of the major olfactory subsystems at the level of a glomerular map. **(A)** Dorso-caudal view of the olfactory bulb (left side) depicting the different glomerular subsystems as indicated by the color-coded legend. Cav2.1 glomeruli in the caudal MOB reside in juxtaposition to the GC-D necklace glomeruli innervated by GC-D+ OSNs of the MOE and the GC-G glomeruli innervated by GGNs of the Grueneberg ganglion (GGN). Cav2.1 glomeruli in the center of the dorsal MOB intermingle with Taar glomeruli, and are also present in the medial MOB. **(B)** Medial view of the right olfactory bulb visualizing the necklace glomerular tracks for GC-D and GC-G, the dorsal position of Cav2.1 and Taar glomeruli, the medial Cav2.1 glomeruli, and the rostral and ventral position of Trpc2+ glomeruli in the MOB. Arrows denote orientations a (anterior), p (posterior), d (dorsal), v (ventral), l (lateral), and m (medial); dashed lines indicate dorsal zones DI and DII, and the ventral zone V. Adapted and extended from Bear et al. (2016).

Our work provides new insight into the architecture, organization, and projection targets of distinct mouse olfactory bulb glomeruli. We showed previously that the vast majority of glomeruli in the MOB and AOB express the N-type calcium channel Cav2.2 and subsequently analyzed its key role in synaptic transmission (Weiss et al., 2014). Here, we identify Cav2.1 as a second candidate for synaptic transmission at the first olfactory synapse within a defined subset of MOB glomeruli. In adult mice, we detected robust Cav2.1 staining in a distinct set of about 20 glomeruli per bulb hemisphere, predominantly located in the medial and central dorsal MOB, and at the dorso-caudal border (see **Figure 9**) whereas no staining was detected in the AOB. Remarkably, the position of Cav2.1 glomeruli in the dorsal center and in the dorso-caudal border of the MOB (**Figure 9**) coincided with two areas harboring known olfactory subsystems that process olfactory cues associated with the detection of threat or danger signals. Notably, 5–7 Cav2.1+ glomeruli resided in juxtaposition to the GC-D+ necklace glomerular subsystem. GC-D+ OSNs use cGMP signaling and are involved in the social transmission of food preference (Leinders-Zufall et al., 2007; Munger et al., 2010). As illustrated in **Figure 9**, the necklace region also contains the glomeruli targeted by GGNs that mediate the detection of cold stimuli (Mamasuew et al., 2008; Schmid et al., 2010) and responses to predator odors and alarm pheromones (Brechbühl et al., 2008, 2013; Pérez-Gómez et al., 2015; Bumbalo et al., 2017). Our results clearly showed that Cav2.1+ glomeruli in the necklace area are distinct from these two non-canonical olfactory subsystems. Instead, Cav2.1+ glomeruli are characterized by the expression of Cnga2 and Pde4a, both known to be essential signaling components of canonical OSNs (Ferguson and Zhao, 2016). Cav2.1+ glomeruli are also clearly distinct from those glomeruli targeted by a subset of OSNs that use the ion channel Trpc2 and the soluble guanylyl cyclase Gucy1b2 to respond to decreases in environmental oxygen (Omura and Mombaerts, 2015; Bleymehl et al., 2016). The necklace glomerular system exhibits extensive intra-glomerular connections with canonical glomeruli (Cockerham et al., 2009) and functional analyses recently suggested that olfactory input from necklace and juxta-positioned canonical glomeruli could be integrated as part of an interconnected bulbar network (Uytingco et al., 2016). Future experiments will be required to determine whether the Cav2.1+ glomeruli are interconnected with other subsystems of the necklace region.

In the dorsal MOB, we detected Cav2.1+ glomeruli in close proximity to glomeruli of the Taar subsystem (Liberles et al., 2009; Pacifico et al., 2012) known to mediate innate fear to predator odors and aversion to spoiled food odors (Kobayakawa et al., 2007; Matsumoto et al., 2010; Dewan et al., 2013). Dorsal Taar+ glomeruli were previously shown to colocalize with Ncam2 (Johnson et al., 2012), an adhesion molecule usually defining ventro-lateral glomeruli that receive axonal input from OSNs located in zones 2–4 of the MOE (Yoshihara et al., 1997). Most of the Cav2.1+ glomeruli were negative for Ncam2 which is typical for OSNs of the dorsal zone 1 in the MOE, but we also detected a small number of Cav2.1+/Ncam2+ glomeruli (∼2–3 out of 20). However, we found no evidence that these glomeruli are positive for Taar expression. Thus, we conclude that Cav2.1+ glomeruli most likely receive axonal input from a subset of canonical OSNs. We note that we cannot fully exclude the possibility that the sensory neurons targeting the Cav2.1+/Ncam2+ glomeruli express a particular type of Taar that could exhibit low affinity to the panTaar antibody used here and thus escaped detection.

Consistent with our results in the MOB, we also found Cav2.1 expression in a defined subpopulation of OSNs of the MOE. In agreement with the positions of Cav2.1+ glomeruli in the MOB, Cav2.1+ OSNs were present in the medial and dorsal aspects of the MOE as verified by two independent techniques, immunohistochemistry and RNAscope *in situ* hybridization. This result is in line with the typical MOE-to-MOB topographic projection patterns observed with OR gene expression (Mori et al., 2006). The scattered distribution of Cav2.1+ OSNs resembled the stochastic patterns known for individual ORs. However, different from the zonal restriction shown for the expression of single OR types to one out of four dorso-ventral zones (Ressler et al., 1994), Cav2.1+ OSNs were present across zones 1–3. This is consistent with the observation that Cav2.1+ glomeruli seem to receive input from both Ncam2- (zone 1 ORs) and Ncam2+ (zones 2–4 ORs) OSNs (Yoshihara et al., 1997). Detailed future molecular analyses will be required to determine whether Cav2.1+ OSNs express one of the known OR genes, or whether they express yet unknown receptor candidates. Such investigations should provide insight into the specific functions that Cav2.1+ OSNs and the corresponding glomeruli could play in olfaction.

At the level of individual Cav2.1+ OSNs, we detected Cav2.1 immunoreactivity in several OSN compartments. Besides presynaptic axon terminals within a given glomerulus, Cav2.1 was also localized to the OSN dendrite, dendritic knob, somata, and proximal axon. This observation may relate to the multiple functions Cav channels exert at various cellular sites (Catterall, 2011). On the basis of this expression pattern, depolarization-induced Ca^2+^ entry through Cav2.1 could influence multiple cellular mechanisms including synaptic release, action potential discharge patterns, activation or regulation of other Ca^2+^-dependent ion channels, regulation of enzymes, and gene expression. Consistent with an essential role in OSN signaling, we found that Cav2.1 staining of OSN somata, dendrites, and dendritic knobs was already evident shortly after birth but absent at embryonic day 18, implying that the somatic function of Cav2.1 establishes with birth and is required throughout life.

In summary, our experiments have identified a previously unrecognized multiglomerular domain in the MOB that is defined by the expression of Cav2.1. These glomeruli and their corresponding sensory neurons may form a novel olfactory subsystem. Important questions that remain to be answered in future experiments are the following: (1) Which receptor candidates are expressed by the Cav2.1+ OSNs? (2) Is there any functional relationship of the Cav2.1 glomeruli to other olfactory subsystems present in the necklace region of MOB? (3) What is the overall role of this presumed subsystem for olfactory processing? (4) And does this novel subdomain also exist in other mammalian species including humans that normally carry a functional *CACNA1A* gene?

In this context, it is important to note that the Cav2.1 channel seems to be more efficient in transmitter release than Cav2.2 (Millan and Sanchez-Prieto, 2002; Ladera et al., 2009). This raises the intriguing question whether the Cav2.1 subsystem could be specialized to detect odor cues that require high-speed processing at the first olfactory synapse. Alternatively, Cav2.1 may also mediate specific forms of synaptic plasticity and contribute to the regulation of synaptic transmission (Mochida, 2018; Nanou and Catterall, 2018). Double-labeling experiments for Cav2.2 and Cav2.1 will have to be performed in the future.

## Author Contributions

MP: study concept and drafting of the manuscript. MP, MT, LO, and EE: data acquisition (immunohistochemistry, confocal microscopy, 3D reconstruction, electron microscopy, RT-PCR). MP, EE, DR-G, CG, and FZ: data interpretation. MP, MT, LO, and D-RG: figure preparation. LO, DR-G, CG, and FZ: revision of the manuscript. All authors take responsibility for data integrity and data analysis accuracy.

## Conflict of Interest Statement

The authors declare that the research was conducted in the absence of any commercial or financial relationships that could be construed as a potential conflict of interest.

## Footnotes

1 www.ncbi.nlm.nih.gov/nuccore/NM_007578.3
